# Tissue Level Changes after Maxillary Sinus Floor Elevation with Three Types of Calcium Phosphate Ceramics: A Radiological Study with a 5-Year Follow-Up

**DOI:** 10.3390/ma14061471

**Published:** 2021-03-17

**Authors:** Wilhelmus F. Bouwman, Nathalie Bravenboer, Christiaan M. ten Bruggenkate, Francis A. Eijsackers, Najada Stringa, Engelbert A. J. M. Schulten

**Affiliations:** 1 Department of Oral and Maxillofacial Surgery/Oral Pathology, Amsterdam Movement Sciences, Amsterdam UMC and Academic Centre for Dentistry Amsterdam (ACTA), Vrije Universiteit Amsterdam, De Boelelaan 1117, 1081 HV Amsterdam, The Netherlands; wbouwman@tergooi.nl (W.F.B.); chris@tenbruggenkate.com (C.M.t.B.); 2 Department of Oral and Maxillofacial Surgery, The Tergooi Hospital, Rijksstraatweg 1, 1261 AN Blaricum, The Netherlands; 3 Department of Clinical Chemistry, Amsterdam Movement Sciences, Amsterdam UMC, Vrije Universiteit Amsterdam, De Boelelaan 1117, 1081 HV Amsterdam, The Netherlands; n.bravenboer@amsterdamumc.nl; 4 Department of Oral and Maxillofacial Surgery, Alrijne Hospital, Simon Smitweg 1, 2353 GA Leiderdorp, The Netherlands; francis@francis4yourpractice.nl; 5 Francis4YourPractice, Julius Caesarlaan 180, 2314 BS Leiden, The Netherlands; 6 Department of Epidemiology and Data Science, Amsterdam UMC—VUmc, Van der Boechorststraat 7, 1081 BT Amsterdam, The Netherlands; n.stringa@amsterdamumc.nl

**Keywords:** biphasic calcium phosphate, beta-tricalcium phosphate, calcium phosphate ceramic, bone substitute, sinus augmentation, sinus floor elevation, radiological measurements

## Abstract

This study evaluates the radiological changes in tissue height after maxillary sinus floor elevation (MSFE) using three types of calcium phosphate ceramics over a period of up to 5 years after dental implant placement. In 163 patients, MSFE was performed. Three groups of patients were distinguished and treated based on the type of calcium phosphate ceramic used and radiologically evaluated: 40 patients with β-tricalcium phosphate (β-TCP), 76 patients with biphasic calcium phosphate (BCP) 20% hydroxyapatite (HA)-80% β-TCP, and 47 patients with BCP 60% HA-40% β-TCP. Radiological measurements were performed on panoramic radiographs at several time points up to 5 years after dental implant placement. After MSFE, a slow decrease in tissue height measured over time was seen in all three study groups. Resorption of the grafted bone substitutes was more prominent in β-TCP than in BCP ceramics with an HA component (60/40 and 20/80). Loss of tissue height after 5 years was lowest in BCP 60/40 and highest in β-TCP. This radiological study shows a predictable and comparable behavior of the slow decrease in tissue height over time for all three types of calcium phosphate ceramics used in MSFE. The fraction of HA in calcium phosphate ceramics and dental implant loading seems to be beneficial for tissue height maintenance after MSFE.

## 1. Introduction

Maxillary sinus floor elevation (MSFE) is a common pre-implant surgical procedure to increase the vertical dimension in the posterior edentulous maxilla in order to place dental implants for oral rehabilitation [1,2,3,4]. For this internal augmentation, the surgeon has the choice between autogenous bone grafts and bone substitutes [5,6,7]. Although autogenous bone grafting, considered to be the gold standard, is a well-known and reliable grafting procedure [8,9], it has the disadvantage of the necessity of a second surgical procedure and, therefore, a higher risk of surgical morbidity and complications [10,11,12,13,14]. For this reason, in some cases a bone substitute is chosen as graft material [12,15]. Different available bone substitutes, such as calcium phosphate ceramics, seem to behave similarly [16,17,18,19,20]. They will be partially replaced in time by vital bone when used in MSFE [5,21]. On the cranial site, the grafted calcium phosphate ceramics should be stable in height as a space maintaining structure to allow new bone to grow into the bone substitute at a later stage and keep the “new” maxillary sinus floor away from the “apical” parts of the dental implants.

Several types of calcium phosphate ceramics have become available with different ratios regarding the crystalline components. Examples of calcium phosphate ceramics are 100% β-tricalcium phosphate (β-TCP), which is osteoconductive and provides a scaffold for the ingrowth of newly formed bone, a biphasic calcium phosphate (BCP) consisting of 20% hydroxyapatite (HA) and 80% β-TCP (BCP 20/80), a BCP consisting of 60% HA and 40% β-TCP (BCP 60/40), or 100% HA (all percentages are weight-based). β-TCP is thought to dissolve much quicker than HA. HA is brittle, rigid and resorbs hardly when used in MSFE, which might hinder replacement of the bone substitute by vital bone [5,22,23,24,25]. The purpose of using a combination of β-TCP and HA is to balance the rate of vital bone ingrowth with the resorption rate of the bone substitute. The choice for β-TCP is in line with the theory that this ceramic would dissolve rather quickly in favor of bone replacement of the dissolved calcium phosphate [6,7,8,19,20,26,27,28]. Nevertheless, it takes substantial time for new bone to invade into the graft material [1,5].

In the literature, several studies evaluated one type of calcium phosphate ceramic β-TCP or β-TCP and HA, in different ratios, mixed with autogenous bone, platelet-rich plasma, or stem cells in MSFE with a study duration ranging from 1 to 9 years [4,29,30,31,32,33]. These studies generally compared the calcium phosphate ceramic with autologous bone, since this is considered the golden standard. However, as yet, volume changes in different ceramics containing different proportions of HA were never compared amongst one another. Comparison of the clinical behavior of different calcium phosphate ceramics without any modifications is important to help the surgeon to choose a suitable bone substitute in maxillary sinus augmentation procedures. To optimize the results of MSFE, it is important to gain insight into the tissue maintenance, resorption patterns, final gain and absolute loss of tissue height, and the specific properties of the available calcium phosphate ceramics with different ratios of HA.

Newly formed bone, especially non-loaded vital bone, is at risk for resorption, resulting in height loss [34]. Therefore, placement of dental implants 6 months after MSFE is important, as dental implants are considered as “loaded pillars” preventing the resorption of bone. A certain degree of vital bone and substitute level dips between the “apices” of dental implants is reported in the literature [35]. This scalloping of the bone (in fact, this is the elevated maxillary sinus floor) would indicate that vital bone resorbs faster in non-loaded areas than in loaded areas [36,37,38].

The aim of this study was to investigate the radiological changes in tissue height after MSFE using three types of calcium phosphate ceramics (β-TCP, BCP 20-80, and BCP 60/40) with a follow-up time of 5 years after dental implant placement. In particular, the following issues were addressed: (1) the initial gain and subsequent loss in tissue height after MSFE, (2) the influence of dental implants on the maintenance of tissue height after MSFE, (3) the difference in tissue height maintenance between the three types of calcium phosphate ceramics used, and (4) the influence of the type of calcium phosphate on the maintenance of tissue height between the measurements at implant site (loaded bone) and distal position (unloaded bone).

## 2. Materials and Methods

### 2.1. Patients

This retrospective study is based on chart reviews (data collection from the medical records of patients) and measurements on panoramic radiographs taken during yearly follow-up visits of patients. In total, 169 patients underwent MSFE as a pre-implant surgical procedure with a calcium phosphate ceramic. Of these 169 patients, 163 were available for follow-up evaluation. In these 163 patients, 256 dental implants were placed. At 4 years follow-up, 81 implant sites were available for investigation, and at 5 years, 43 implant sites were available for radiological measurements. The criteria for treatment selection was a minimal native alveolar bone height of minimal 4 mm in the posterior maxilla and the patient’s wish for prosthetic rehabilitation with dental implants. Patients with a history of radiation of the jaws, patients with heart valve prostheses and a history of endocarditis and patients who had a MSFE with simultaneous lateral augmentation of the posterior maxilla were not included in this study. Smokers (21%) were not excluded. All patients received standard care and signed a written consent prior to both surgical procedures (MSFE and dental implant surgery) for the use of their data.

One hundred and sixty-three patients (75 males, 88 females, age ranging from 18 to 78 years) were divided in three groups based on the type of bone substitute used: β-TCP (Ceros^®^, Thommen Medical, Grenchen, Switzerland), BCP 20/80 (Institut Straumann AG, Basel, Switzerland), BCP 60/40 (Straumann^®^ Bone Ceramic (SBC), Institut Straumann AG, Basel, Switzerland).

Ceros TCP granules had a total porosity of 60% with interconnecting macropores of 100–500 μm and a particle size of 700–1400 μm. The two BCPs had similar particle size (500–1000 μm), microporosity (2%), interconnected pores (100–500 μm), and porosity (90%). BCP 20/80 had a crystal size of 1.0–6.0 μm and a specific surface area of 9.5 × 10^−3^ m^2^/g, while BCP 60/40 had a crystal size of 0.6–6.0 μm and a specific surface area of 6.9 × 10^−3^ m^2^/g. Table 1 shows an overview of the physical properties of three types of calcium phosphate ceramics. All calcium phosphate ceramics were used as received without any modifications.

The group treated with β-TCP consisted of 40 patients (21 males, 19 females; mean age: 57 years; range: 27 to 78 years), the group treated with BCP 20/80 consisted of 76 patients (32 males, 44 females; mean age: 59 years; range: 18 to 79 years), and the group treated with BCP 60/40 consisted of 47 patients (22 males, 25 females; mean age: 55 years; range 18 to 77 years). Patient data are shown in Table 2.

### 2.2. Maxillary Sinus Floor Elevation Procedure

The MSFE was performed according to Tatum’s “top-hinge-trapdoor-technique” under local anesthesia [3]. Patients received an antibiotic prophylaxis, amoxicillin 500 mg, 4 times daily (or in case of allergy, clindamycin 300 mg 4 times daily) for 7 days, starting the day before MSFE. For oral hygiene, chlorhexidine digluconate 0.12% (*w*/*w*) 2 times daily (10 mL) for 2 weeks was prescribed, according to the manufacturers’ instructions. A mid-crestal incision was made as surgical flap design with mesial and distal buccal and vertical release incisions. In the three groups, the created area at the maxillary sinus bottom was filled with the selected graft material for that group of patients. No membrane was used to cover the lateral window [39]. The wounds were closed with Gore-Tex^®^ sutures (W.L. Gore & Associates, Newark, DE, USA). After 10 to 14 days the sutures were removed.

### 2.3. Dental Implant Placement

The 256 Straumann^®^ SLA Soft Tissue Level dental implants with a diameter of 3.3, 4.1, or 4.8 mm and implant length of 10 or 12 mm were placed under local anesthesia 6 months after MSFE, according to the manufacturers’ instructions (Institut Straumann AG, Basel, Switzerland). The wounds were closed with Gore-Tex^®^ sutures. All patients received a prophylactic dose of 3 g amoxicillin 1 h prior to dental implant surgery (or in case of an allergy, clindamycin 600 mg). The dental implants were left to integrate in a non-submerged unloaded fashion.

For oral hygiene chlorhexidine digluconate 0.12% (*w*/*w*), 2 times daily 10 mL for 2 weeks was prescribed, according to the manufacturers’ instructions, as part of the standard procedure for dental implant surgery. To allow postoperative radiological evaluation, a panoramic radiograph (Orthophos XG, Sirona Dental Systems GmbH, Bensheim, Germany) was taken immediately after dental implant placement. Removal of the sutures was performed 10 to 14 days after dental implant surgery. Three months after dental implant placement, prosthetic treatment was started by a restorative dentist.

### 2.4. Radiological Evaluation

**Figure 1:**
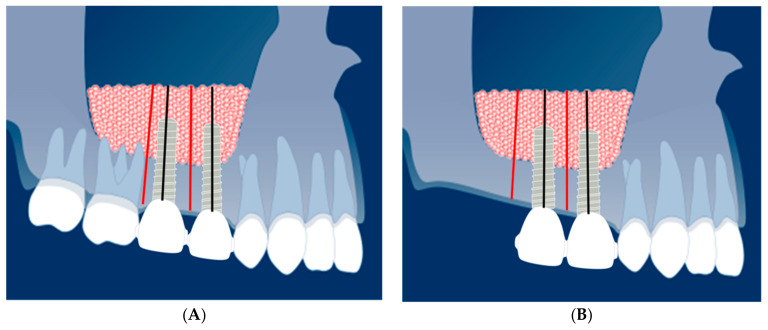
(**A**) This graphic shows the tissue height measurements at inter-implant positions. The black lines indicate the measurement positions at implant sites; the red lines (2–3 mm distally) the “distal” or intermediate positions. (**B**) This graphic shows the tissue height measurements at distal positions (free-end situation). The black lines indicate the measurement positions at implant sites; the red lines (2–3 mm distally) the “distal” positions. Source: graphic used with courtesy of the ITI Foundation, Basel, Switzerland.

Panoramic radiographs were taken at patient intake, approximately 2 months prior to MSFE (T0), immediately after MSFE (T1), and 4 weeks prior to dental implant surgery ridge mapping is performed, which is a measurement procedure to ensure that the diameter of a dental implant does not exceed the dimensions of available bone (T2), immediately after dental implant placement (T3), at the end of the integration period, after prosthetic loading (T4), and later during the yearly recall visits (T5–T9). On these panoramic radiographs, changes in tissue height of the grafted area were measured at the implant site (black line) and 2–3 mm distally of the dental implant site (red line) (Figure 1A,B). All panoramic radiographs were taken with an average magnification of 1.25. The measured tissue height on the panoramic radiographs was multiplied with 0.8 to obtain the true height in mm.

### 2.5. Statistical Analysis

Data are expressed as mean plus or minus standard deviation. Since the heights were measured several times (T0–T9) in the same individual and 256 implants were measured in 163 patients, a mixed model analysis (T0–T9) was applied to adjust for the non-independence in the data. Correction/adjustment was performed by estimating the variance of the intercepts (random intercepts). The effect modification was checked by estimating the variance of the slope (random slope). Maximum height loss was calculated at T8 and tested using an ANOVA. *p* < 0.05 was considered significant. IBM SPSS version 26 was used for all statistical analyses.

## 3. Results

### 3.1. Patient Data

Patient data of all 512 sites (256 implant sites and 256 distal positions) in 163 patients after MSFE with calcium phosphate ceramics were gathered and combined. In Table 2, the demographic data of the study patients and the numbers and types of calcium phosphate ceramics used as a graft material in MSFE are shown.

In Table 3, the number of measurements that could be performed based on the availability of radiological data are shown.

### 3.2. Maxillary Sinus Floor Elevation with Calcium Phosphate Ceramics

Tissue levels (163 patients at 256 implant sites and 256 distal positions) were measured on panoramic radiographs and calculated in millimeters (Figure 1A,B). Table 4 shows the data from the “combined measurements” and “the individual measurements” of the three calcium phosphate ceramic groups.

### 3.3. Comparison of Three Individual Types of Calcium Phosphate Ceramics

**Figure 2:**
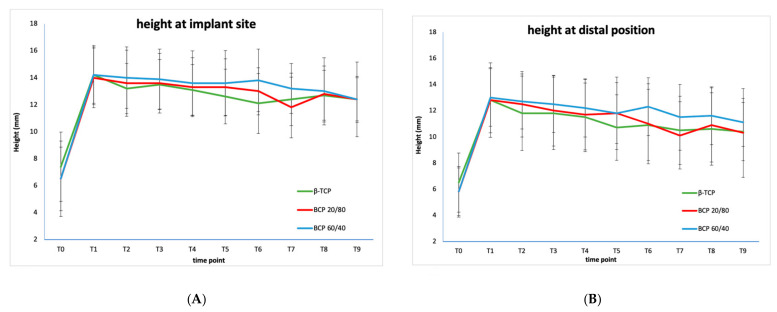
Mean tissue height changes at implant site (**A**) and distal position (**B**) measured (in mm) over a period of 5 years. T0 (−8): patient intake, T1 (−6): maxillary sinus floor elevation, T2 (−1): ridge mapping, T3 (0): dental implant placement, T4 (3): prosthetic loading, T5 (12): follow-up visit after 1 year, T6 (24): follow-up visit after 2 years, T7 (36): follow-up visit after 3 years, T8 (48): follow-up visit after 4 years, T9 (60): follow-up visit after 5 years. β-TCP: β-tricalcium phosphate; BCP: biphasic calcium phosphate; HA: hydroxyapatite.

The mean tissue heights of the three individual different types of calcium phosphates are shown in Table 4 and Figure 2.

The initial tissue height gain by means of MSFE, calculated at T1, is comparable in all three groups (β-TCP 6.5 mm, BCP 20/80: 7.2 mm, BCP 60/40: 7.4 mm). The mixed model analysis revealed no significant difference in the height distribution over time between β-TCP, BCP 20/80, and BCP 60/40 at the implant site. At the distal position, however, a significant difference was observed between β-TCP and BCP 20/80 (*p* = 0.002), but not between β-TCP and BCP 60/40 (*ns*). The final tissue height loss at the implant site, calculated at T8 and T9, was the highest in the β-TCP group and the lowest in the 60/40 group (β-TCP: 1.5 mm and 1.8 mm, BCP 20/80: 1.2 mm and 1.6 mm and BCP 60/40: 1.2 mm and 1.8 mm, respectively), although this trend was not significant for both time points. The final tissue height loss at the distal positions, however, was significantly different at T8 (ANOVA: *p* = 0.012). A post-hoc analysis showed final tissue height loss was lower in the BCP 60/40 compared to β-TCP group (*p* = 0.017) and compared to the BCP 20/80 group (*p* = 0.029). Final tissue height loss was also lower in the BCP 20/80 group compared to the β-TCP group (*p* = 0.018).

### 3.4. Comparison of the Combined Calcium Phosphate Ceramics at Implant Position and Inter-Implant (Distal) Position

The pattern of the mean tissue height changes of the combined calcium phosphate ceramics at two positions: at dental implant site and distally of the implant site (Figure 1A,B). These “distal” positions can be regarded as inter-implant or inter-implant-tooth positions or, in case of a free-ending situation, as distal positions. Table 4 shows the pattern of mean tissue height changes in the posterior (partially) edentulous atrophic maxilla after MSFE surgery with a calcium phosphate bone substitute. The maxillary sinus with limited alveolar crest height (initial height 6.5 mm = T0) is internally augmented with a bone substitute 6 months prior to dental implant placement (13.5 mm = T1). Therefore, a mean increase in tissue height is 7.0 mm immediately after MSFE. The tissue height change showed a decrease of 0.6 mm (after MSFE) at the time of dental implant placement (12.9 mm = T3). Over time, the tissue height diminishes further to 2.0 mm 5 years after MSFE (T9). The total tissue height gain after 5 years is 5.0 mm, from 6.5 mm (T0) resulting in a final tissue height of 11.5 mm (T9). The final tissue height loss is 1.6 mm at 4 years and 2.0 mm at 5 years after dental implant placement. The pattern of tissue height loss at the two positions appears to be the same but the mean tissue height loss at dental implant position is 1.3 mm at 4 years and 1.7 mm at 5 years. At distal position the tissue height loss is 1.8 mm and 2.3 mm at 4 and 5 years after dental implant placement, respectively.

**Figure 3:**
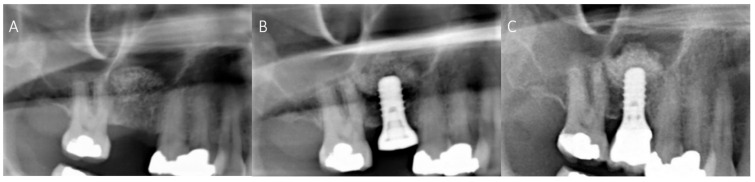
Radiographs of the right partial edentulous maxilla after maxillary sinus floor elevation (**A**), after dental implant placement (**B**) and at 2-year follow-up (**C**). Apical to the dental implant the graft material has been lifted upwards, resulting in a higher local tissue height at the implant position.

On several panoramic radiographs a particular phenomenon was found, indicating that apically of the inserted dental implant some of the graft material appeared to have been lifted in the cranial direction (“Summers” effect/method) [40]. The tissue height at implant site increased after MSFE. Figure 3A–C show radiographs with clear signs of this tissue height increase. This postoperative increase in height can also be recognized in Table 4 at timepoint T3.

## 4. Discussion

In this study, the radiologically derived tissue height distribution following MSFE with three types of calcium phosphate ceramics were compared over a period of 5 years after dental implant placement. In all cases, the use of calcium phosphate ceramics in MSFE resulted in an initial tissue height gain followed by an initial decrease in tissue height which subsides after dental implant placement. This pattern is consistent with previously reported MSFE procedures using bone substitutes [5,28,30,41,42], also reported in the meta-analyses by Haugen et al. [17].

Apart from the three fully synthetic calcium phosphate bone substitutes used in the present study, xenografts, such as demineralized bovine bone mineral (DBBM), have been also widely used in MSFE procedures [43,44,45,46,47]. Clinical, radiological, and histomorphometrical studies on DBBM confirmed predictable outcomes after MSFE, meaning good quality and volume of regenerated tissue [17]. DBBM has a chemical composition similar to human bone, being osteoconductive and showing a slow resorption rate comparable to 100% HA, resulting in stabilization of the grafted material and a high dental implant survival rate [48,49,50,51,52]. Several studies compared DBBM to 100% β-TCP and/or synthetic BCPs composed of different ratios of HA and β-TCP [29,51,53,54,55,56,57]. Overall, MSFE procedures with synthetic BCPs (HA/TCP) and DBBM seem to exhibit similar results, regarding new bone formation and the survival of dental implants. However, studies on volume stability after MSFE, comparing various calcium phosphate ceramics containing different proportions of HA and β-TCP, are scarce in the literature [29,55,58]. In this respect, no studies have been reported comparing DBBM to 100% β-TCP or 60/40 or 20/80 mixtures of HA and β-TCP.

Height loss (after MSFE) may be due to settling (or clinging) of the calcium phosphate granules in the maxillary sinus and at a later stage resorption of the grafted material. At the same time, height loss of grafted material due to air pressure from respiration in the maxillary sinus is also unavoidable [59,60].

In all three groups, the loss of tissue height at the distal positions was more prominent than at the dental implant site. This could have a mechanical explanation [61,62], since dental implants can be considered as stable pillars, which eventually may lead to some degree of scalloping. Another explanation for the height differences between implant sites and distal positions could be that the calcium phosphate granules are pushed up at implant sites during the osteotomy and placement of the dental implants at dental implant surgery or by an ossifying hematoma [63]. This can be regularly observed at postoperative radiographs after implant placement [34,38,41].

The height of vital bone within the total tissue volume is the relevant structure for dental implant stability [21,64]. This vital bone consists of the original alveolar bone and the newly formed bone between the bone substitute granules as a result from osteoconduction in cranial direction. As resorption of β-TCP seems to occur at a higher rate than HA, the question is whether the resorption results in replacement by vital bone that is supposed to connect with the titanium implant root surface, eventually resulting in osseo-integration of the dental implant. From the literature, there is little or no support for this. This is the reason why for augmentation purposes BCPs have been developed [6,29,52,65].

A recent micro-CT study by Helder et al. [66] shows BCP 20/80 might perform better, at least in the short term, as a scaffold for bone augmentation in the MSFE model than BCP 60/40 as more bone is formed, and more osteoid is deposited at the cranial side in BCP 20/80 treated patients compared to BCP 60/40 treated patients.

The term “tissue height” is used instead of “bone height” in this study, since we know that, in spite what some commercial companies tend to promote, the transition from a bone substitute to vital bone is a very slow and incomplete process [5,62].

Many publications refer to radiological bone height after sinus floor elevation, actually meaning tissue height, consisting of three layers of tissues: native bone from the original alveolar crest, a transition layer consisting of a mixture of bone substitute and new vital bone, and a cranial layer of remaining bone substitute [4,5,7]. As the process of bone generation in the grafted area is slow, time plays an important role. Six months healing time after MSFE generates approximately 3 mm of bone gain [41]. This means that a substantial native bone height is required for primary stability of the placed dental implants, even after a 6-months healing period. In fact, from our former study, it can be concluded that a 9-month healing period after MSFE using BCP 60/40 would be favorable [5].

Less than 4 mm native bone height does not seem to be the suitable indication for the use of bone substitutes. [7] A further complication could be if the cranially situated bone substitute resorbs, without turning into vital bone in time. If this bone substitute dissolves, the apical parts of the dental implants would become dehiscent. In the present study, this was not observed over a period of 5 years after dental implant placement. Although the “insolvable” hydroxyapatite component in the calcium phosphate bone substitutes appears to maintain the graft on the cranial side best, it can be regarded as a covering or protective layer of tissue to cover the apical parts of the dental implant from exposing into the elevated maxillary sinus, without giving support to the dental implant. One could regard this layer of mainly calcium phosphate and fibrous tissue as merely a shielding structure for the “new” maxillary sinus.

This study also has some limitations. One limitation is the missing values in the 5-year follow-up. As a result, the consistent trend in final tissue height loss did not show a significant difference at T9. For this reason, final tissue height loss was also calculated at T8, 4 years after the dental implantation. At this time point significant differences were found at the distal position. Nevertheless, studies with a 4 to 5-year follow-up are rare but our study provided valuable insights in MSFE, dental implant placement, and final height loss of different calcium phosphate ceramics.

At the time of surgery, 25.7% of the Dutch population was smoking. As the negative influence of smoking for dental implants was known, our patients were advised to stop smoking if they did. This resulted in a percentage of smokers of 21% in our patient population. However, the aim of this study was to evaluate the changes in tissue height in a certain patient population, not the dental implant survival rate or the influence of risk factors.

## 5. Conclusions

This radiological study shows a predictable and comparable behavior of slow decrease in tissue height over time for all three types of calcium phosphate bone substitutes used in MSFE. Mean tissue height was better maintained at implant sites than at inter-implant or inter-implant-tooth positions or free-end-positions. The use of β-TCP results in a greater tissue height loss, compared to the use of HA-containing calcium phosphate ceramics. The fraction of HA in calcium phosphate ceramics in combination with dental implant loading seems to be beneficial for tissue height maintenance after MSFE.

## Figures and Tables

**Table 1:** Physical properties of three types of calcium phosphate ceramics, as specified by the manufacturers. β-TCP—β-tricalcium phosphate; BCP—biphasic calcium phosphate; HA—hydroxyapatite.

	β-TCP	BCP 20/80	BCP 60/40
Porosity	60%	90%	90%
Interconnecting macropores	100–500 μm	100–500 μm	100–500 μm
Particle size	700-1400 μm	500–1000 μm	500–1000 μm
Microporosity (vol%)	1–2%	2%	2%
Crystal size	Not specified.	1.0–6.0 μm	0.6–6.0μm
Specific surface area	Not specified.	9.5 × 10^−3^m^2^/g	6.9 × 10^−3^m^2^/g

**Table 2:** Patient data of the three types of calcium phosphate ceramic. β-TCP—β-tricalcium phosphate; BCP—biphasic calcium phosphate; HA—hydroxyapatite; M—male; F—female; MSFE—maxillary sinus floor elevation.

	β-TCP	BCP 20/80	BCP 60/40
Number of patients included	40	76	47
Number of implant sites included	69	120	67
M/F	21 M, 19 F	32 M, 44 F	22 M, 25 F
Mean age in years	57	59	55
Age range in years	27–78	18–79	18–77
MSFE performed between	2/2009–6/2012	3/2010–9/2012	1/2009–11/2013

**Table 3:** Number of measurements available at several time points up to 5-year follow-up. β-TCP—β-tricalcium phosphate; BCP—biphasic calcium phosphate; HA—hydroxyapatite; T0—patient intake; T1—maxillary sinus floor elevation; T2—ridge mapping; T3—dental implant placement; T4—prosthetic loading; T5—follow-up visit after 1 year; T6—follow-up visit after 2 years; T7—follow-up visit after 3 years; T8—follow-up visit after 4 years; T9—follow-up visit after 5 years.

	Months of Observation	β-TCP	BCP 20/80	BCP 60/40	Total
T0	−8	40	76	47	163
T1	−6	40	76	47	163
T2	−1	40	76	47	163
T3	0	40	76	47	163
T4	3	40	38	47	125
T5	12	35	21	26	82
T6	24	17	34	27	78
T7	36	23	28	15	66
T8	48	29	30	22	81
T9	60	25	8	10	43

**Table 4:** Individual and combined tissue height measurements (in mm) of 512 sites (256 implant site and 256 distal positions) of three types of calcium phosphate bone substitutes in maxillary sinus floor elevation (MSFE), after 5-year follow-up. β-TCP—β-tricalcium phosphate; BCP—biphasic calcium phosphate; HA—hydroxyapatite; T0—patient intake; T1—MSFE; T2—ridge mapping; T3—dental implant placement; T4—prosthetic loading; T5—follow-up visit after 1 year; T6—follow-up visit after 2 years; T7—follow-up visit after 3 years; T8—follow-up visit after 4 years; T9—follow-up visit after 5 years.

Time Points	Months of Observation	β-TCP			BCP 20/80			BCP 60/40			Combined Calcium Phosphates		
		Implant Site Height mm (mean ±SD)	Distal Position Height mm (mean ±SD)	Mean Tissue Height mm	Implant Site Height mm (mean ±SD)	Distal Position Height mm (mean ±SD)	Mean Tissue Height mm	Implant Site Height mm (mean ±SD)	Distal Position Height mm (mean ±SD)	Mean Tissue Height mm	Mean tissue Height Implant Site mm	Mean Tissue Height Distal Site mm	Mean Tissue Height mm
T0	−8	7.4	6.5	7.0	6.5	5.8	6.2	6.5	5.8	6.2	6.7	6.2	6.5
		± 2.8	± 2.3	± 2.6	± 2.4	± 1.8	± 2.1	± 2.6	± 1.9	± 2.3	± 2.6	± 2.0	± 2.3
T1	−6	14.2	12.8	13.5	14.0	12.8	13.4	14.2	13.0	13.6	14.1	12.8	13.5
		± 2.2	± 2.8	± 2.6	± 2.2	± 2.5	± 2.4	± 2.1	± 2.2	± 2.2	± 2.2	± 2.5	± 2.4
T2	−1	13.2	11.8	12.5	13.6	12.5	13.1	14.0	12.7	13.4	13.5	12.4	13.0
		± 2.4	± 2.8	± 2.7	± 2.3	± 2.5	± 2.4	± 1.9	± 2.1	± 2.1	± 2.2	± 2.5	± 2.4
T3	0	13.5	11.8	12.7	13.6	12.0	12.8	13.9	12.5	13.2	13.6	12.1	12.9
		± 2.2	± 2.8	± 2.6	± 2.2	± 2.7	± 2.6	± 1.8	± 2.2	± 2.1	± 2.1	± 2.6	± 2.5
T4	3	13.1	11.5	12.3	13.3	11.7	12.5	13.6	12.2	12.9	13.3	11.8	12.6
		± 2.2	± 2.6	± 2.5	± 2.4	± 2.7	± 2.7	± 1.9	± 2.2	± 2.2	± 2.2	± 2.6	± 2.5
T5	12	12.6	10.7	11.7	13.3	11.8	12.6	13.6	11.8	12.7	13.2	11.4	12.3
		± 2.1	± 2.5	± 2.5	± 2.4	± 2.8	± 2.7	± 2.0	± 2.3	± 2.3	± 2.2	± 2.7	± 2.6
T6	24	12.1	10.9	11.5	13.0	11.0	12.0	13.8	12.3	13.1	13.0	11.4	12.2
		± 1.7	± 2.7	± 2.3	± 2.3	± 3.1	± 2.9	± 2.2	± 2.2	± 2.3	± 2.2	± 2.7	± 2.6
T7	36	12.4	10.5	11.5	11.8	10.1	11.0	13.2	11.5	12.4	12.6	10.8	11.8
		± 2.3	± 2.6	± 2.6	± 1.9	± 2.6	± 2.4	± 2.0	± 2.5	± 2.3	± 2.0	± 2.6	± 2.5
T8	48	12.7	10.6	11.7	12.8	10.9	11.9	13.0	11.6	12.3	12.8	11.0	11.9
		± 2.1	± 2.8	± 2.6	± 2.5	± 2.8	± 2.8	± 1.8	± 2.2	± 2.1	± 2.1	± 2.6	± 2.6
T9	60	12.4	10.4	11.4	12.4	10.3	11.4	12.4	11.1	11.8	12.4	10.5	11.5
		± 1.6	± 2.2	± 2.2	± 2.8	± 3.4	± 3.1	± 1.7	± 1.8	± 1.8	± 1.8	± 2.3	± 2.3

## Data Availability

Not applicable.

## Abbreviations

BCP	biphasic calcium phosphate
β-TCP	β-tricalcium phosphate
g	gram
HA	hydroxyapatite
ns	not significant
mg	milligram
mL	milliliter
mm	millimeters
MSFE	maxillary sinus floor elevation
*p*-value	null hypothesis significance testing
SBC	Straumann^®^ Bone Ceramic
SD	Standard Deviation ±
SLA	sand-blasted, large-grit, acid-etched
*w/w*	weight-based
